# Bowel Ischemia from Heat Stroke: A Rare Presentation of an Uncommon Complication

**DOI:** 10.1155/2016/5217690

**Published:** 2016-10-20

**Authors:** Umair Masood, Anuj Sharma, Wajihuddin Syed, Divey Manocha

**Affiliations:** ^1^Department of Internal Medicine, SUNY Upstate Medical University, 750 East Adams Street, Syracuse, NY 13210, USA; ^2^Department of Gastroenterology, SUNY Upstate Medical University, 750 East Adams Street, Syracuse, NY 13210, USA

## Abstract

A healthy 27-year-old female presented to the hospital after she collapsed an hour into her first marathon run on a hot humid day. On presentation, she was hyperthermic, encephalopathic, tachycardic, and hypotensive. On admission, she was found to have lactic acidosis, rhabdomyolysis, and acute kidney injury and was treated with cold normal saline and cooling blankets. She subsequently started having abdominal pain and bloody bowel movements. Computed tomography of the abdomen revealed ascending colon thickening. Furthermore, her lab findings showed transaminitis and elevated coagulation parameters. Due to the acute hypotensive state from the heat stroke, patient had developed bowel ischemia, ischemic hepatitis, and disseminated intravascular coagulation, all of which are uncommon complications of heat stroke. She was managed aggressively with intravenous fluid hydration with resolution of her symptoms over the course of 4 days. In addition to the uncommon complications, early presentation of this bowel ischemia despite adequate hydration in such a healthy individual is another unique aspect of the case.

## 1. Introduction

Exertional heat stroke (EHS) is one of the leading causes of death in young athletes [1]. This syndrome is commonly seen in young individuals who engage in heavy exercise during periods of high ambient temperature. We report a case of a young female who developed bowel ischemia and ischemic hepatitis after a marathon run on a warm summer afternoon.

## 2. Case

**Figure 1:**
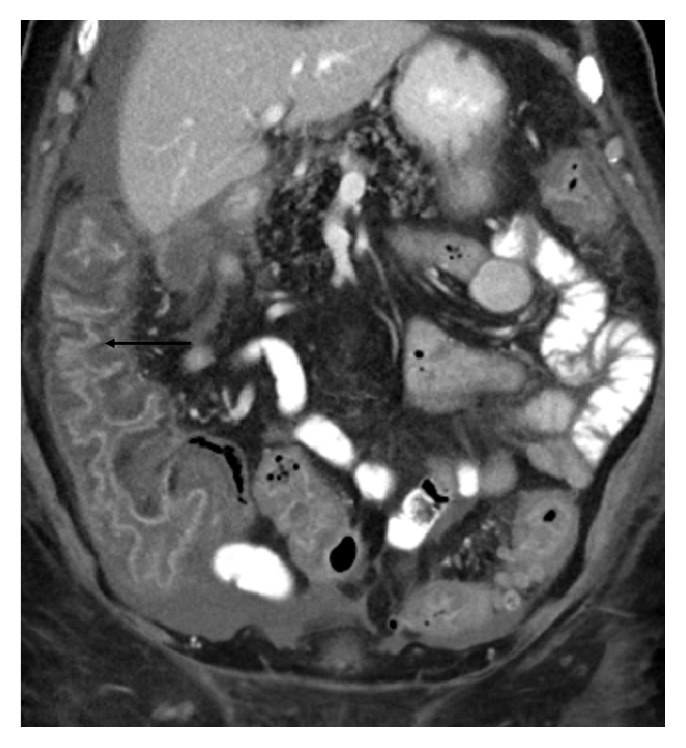
Sagittal view of CT scan of the abdomen showing ascending colon thickening (arrow).

A 27-year-old female was brought to the hospital when she collapsed during her first marathon, an hour into the run. Her friends threw some water on her face to no avail. Emergency medical service was called and patient was immediately brought to the hospital. At presentation, she was severely encephalopathic. Her vitals were pertinent for a temperature of 40.2°C, blood pressure of 82/54 mmHg, and a heart rate of 125 bmp. Her BMI was around 23.29. Laboratory work-up revealed a creatinine of 1.6 mg/dL, potassium of 5.6 mg/dL, lactate of 2.5 mmol/L, and creatine kinase (CK) of 1825 U/L. A computed tomography (CT) scan of the head was negative for any acute disease. She was treated with intravenous (IV) infusion of cold normal saline, cooling blankets, and ice packs with improvement in her mental status. The next day, patient developed abdominal pain and bloody diarrhea with a lactate of 3.6 mmol/L. She did report general intermittent use of ibuprofen but not recently. CT scan of the abdomen revealed ascending colon wall thickening signifying ischemic colitis (Figure 1). Further work-up revealed acute liver injury with elevated transaminases (ALT of 1484 U/L and AST of 1217 U/L) and worsening of rhabdomyolysis with further elevation of CK (2213 U/L). Patient's course was further complicated by disseminated intravascular coagulation (DIC) evident from a platelet count of 92000/*μ*L, d-dimer of 4.2 mcg/mL, fibrinogen of 98 mg/d, prothrombin time of 21 seconds, and partial thromboplastin time of 48 seconds. She was managed with aggressive IV fluid resuscitation for 3 days with normalization of creatinine and lactate and significant improvement in her liver function tests and coagulation parameters. She was discharged after a 4-day hospital course and seen in clinic after 2 weeks with normalization of the rest of her lab abnormalities.

## 3. Discussion

Exertional heat illness (EHI) is a continuum of illnesses relating to the body's inability to cope with heat during physical exertion which range from minor illnesses like heat cramps to life-threatening syndrome known as exertional heat stroke (EHS). EHI is one of the leading causes of death in young athletes in the United States [1]. In addition to athletes, military personnel and laborers performing intense exercise are also at increased risk [1]. The most common risk factors that predispose to any form of EHI include strenuous exercise in high ambient temperature and humidity, dehydration, obesity, poor physical fitness, and lack of acclimatization [2]. In order to understand EHI, understanding the physiology behind regulation of body temperature is essential. Body temperature is regulated in the anterior hypothalamus and is maintained around 37°C ± 1° [3]. During exercise, the human body uses mechanisms of evaporation, radiation, convection, and conduction to dissipate heat [3]. All these mechanisms require increase in shunting of blood to the skin during exercise. During high heat loads when ambient temperature is higher than body's core temperature, conduction, convection, and radiation are not effective. In addition, supply of blood to the skin is limited during exercise as body attempts to fulfill the demands of active skeletal muscles. Evaporative cooling which is the most affective mode of heat dissipation is limited in highly humid conditions due to lack of water vapor pressure gradient [3]. All these aspects contribute towards failure of heat dissipation and the resultant EHI. In this particular case, the marathon reportedly started around 10 am when the ambient temperature was 26.8°C and the humidity was 80%. The estimated wet-bulb globe temperature was 29°C. All these factors contributed towards the increased risk of EHS in our patient.

As mentioned, EHI has a wide spectrum of presentation ranging from heat cramps to exertional heat stroke (EHS). ESH is a multisystem, life-threatening syndrome characterized by central nervous system dysfunction and end organ damage in association with very high body temperatures [3]. The complications of EHS are a direct result of ischemia and oxidative and nitrosative stress which commonly include cerebral ischemia, kidney injury, liver injury, and rhabdomyolysis as seen in our patient. CNS dysfunction can manifest as a wide range of possible symptoms and signs, from disorientation and headache to encephalopathy, coma, and seizure [4]. DIC is a an uncommon complication of EHS [5, 6]. Gastrointestinal (GI) complications such as bowel ischemia are also not very common. Bowel ischemia manifested in our patient as GI bleeding. Such complication can occur due to the diversion of blood from the mesenteric vasculature to the skin and muscle during exertion and can be further exacerbated by prolonged hypovolemic state [7]. The fact that our patient presented so acutely despite prompt adequate hydration is very unique. Though she mentioned training prior to presentation, the fact that it was her first marathon run coupled with severe weather condition could have made her prone to such severe presentation.

Treatment of EHS revolves around rapid cooling in addition to supportive measurements. Ice water emersion is generally preferred unless unavailable [8]. Alternative methods of cooling include cold water shower or hose, wet towels, or using a tarp to sheet covered with ice. In our case, ice water emersion was not available immediately; hence alternative modes such as cold saline, cooling blankets, and ice packs were effectively utilized. Cold saline is a current area of active research and literature on use of cooling blankets is conflicting [9].

In conclusion, clinicians and athletes need to be aware of the basic physiologic principles of thermoregulation and the spectrum of heat illness in order to recognize them and allow for timely intervention. Prompt delivery of rapid cooling, adequate fluid resuscitation, and correction of electrolytes derangements is essential to prevent worsening of life-threatening complications such as EHS.
